# Homæopathy in the City of New-York

**Published:** 1847-02

**Authors:** 


*Homœopathy in the City of New-York.*—

"See ocean into tempest tost,
To waft a feather or to drown a fly."

The homœopathics in New-York are in a fair way to devour each other, after the manner of the Kilkenny cats. At their late convocation, it was ascertained that their prominent and conspicuous partizans, including their acknowledged leader (now that Dr. Gram is no more,) had been bleeding, puking, purging, blistering, and mercurializing their patients, while sailing under the Hahnemann flag, and even alternating all these remedies with pellets of sugar of milk, and infinitesimal dilutions. It was in vain that these latter claimed that these allopathic remedies were "homœopathic to the disease," and therefore lawful ammunition in the "work of death;" the honest men who have been duped into the silly conceit that "the doses of drugs cannot possibly be too small," as taught by their great German master, and that the higher dilutions "if shaken from above downward" develop potences, which will "cure all incurable diseases," regarding the "tricks of the trade" to which many of their brethren were addicted, as forfeiting their claims to be recognized as real Simon Pures. But on a trial of numerical strength in the election of officers, it was found that one of the deserters from the pure faith was elected Corresponding Secretary, the most important office in the sect. Whereupon twenty or more, who claim to be pure homœopathics, in contradistinction to those who run an accommodation line, which takes in all sorts of passengers, forthwith repudiated all connection with the society. Another society is to be formed, from which all are to be excluded who do not strictly adhere in all cases to the sugar pellets, moistened with "the infinitesimal solution of nothing." Each of these divisions of the homœopathic army now open a battery on each other, and the result cannot be doubtful, with such redoubtable heroes as Gray and Hull on one side, and Kirby and Snow on the other.

Meanwhile the days of Hahnemann are numbered in this city, for all confidence in its disciples has departed. Its greatest dupes now only take the pellets of sugar of milk, when there is nothing the matter, and whenever they are sick, they abjure homœopathic trifling, and employ a physician proper, who has maintained his integrity in the profession. It it this that has driven many of their practitioners to proffer their patients a resort to allopathy, when they will no longer trust to homœopathy, they professing to practise on either system, or both, to suit the times. This device may serve the purpose of those who have a smattering of knowledge in the old school, but those who know nothing but homœopathy are in a bad fix. *Hanc illæ lachrymæ!* Some of them have taken hold of hydropathy, others of mesmerism, and one of them has become a new recruit to chrono-thermalism, being No. 2 of this new sect. Shade of Hahnemann! *O tempora! O mores!—N. Y. Correspondent of Boston Med. and Surg. Journal.*

